# The application of autofluorescence system contributes to the preservation of parathyroid function during thyroid surgery

**DOI:** 10.1007/s00423-024-03256-5

**Published:** 2024-03-14

**Authors:** XianBiao Shi, Guan Lv, JiaBo Qin, Yixuan Li, Lulu Zheng, Haoran Ding, JianFeng Sang

**Affiliations:** 1 Nanjing Drum Tower Hospital, Nanjing, China; 2 Nanjing Drum Tower Hospital Clinical College of Nanjing Medical University, Nanjing, China; 3 Nanjing Medical University, Nanjing, China; 4 Nanjing University, Nanjing, China

**Keywords:** Thyreoidectomy, Central lymph node dissection, Hypoparathyroidism, Autofluorescence

## Abstract

**Purpose:**

The purpose of this study was to investigate the impact of autofluorescence technology on postoperative parathyroid function and short-term outcomes in patients undergoing thyroid surgery.

**Methods:**

A total of 546 patients were included in the study, with 287 in the conventional treatment group and 259 in the autofluorescence group. Both groups underwent central lymph node dissection, which is known to affect parathyroid function. Short-term outcomes, including rates of postoperative hypocalcemia and parathyroid dysfunction, serum calcium and PTH levels on the first postoperative day, as well as the need for calcium supplementation, were analyzed. A multivariable analysis was also conducted to assess the impact of autofluorescence on postoperative parathyroid dysfunction, considering factors such as age, BMI, and preoperative calcium levels.

**Results:**

The autofluorescence group demonstrated significantly lower rates of postoperative hypocalcemia and parathyroid dysfunction compared to the conventional treatment group. The autofluorescence group also had better serum calcium and PTH levels on the first postoperative day, and a reduced need for calcium supplementation. Surprisingly, the use of autofluorescence technology did not prolong surgical time; instead, it led to a shorter hospitalization duration. The multivariable analysis showed that autofluorescence significantly reduced the risk of postoperative parathyroid dysfunction, while factors such as age, BMI, and preoperative calcium levels did not show a significant correlation.

**Conclusion:**

This study provides evidence that autofluorescence technology can improve the preservation of parathyroid function during thyroid surgery, leading to better short-term outcomes and reduced postoperative complications. The findings highlight the potential of autofluorescence as a valuable tool in the management of parathyroid hypofunction. Further research and validation are needed to establish the routine use of autofluorescence technology in the thyroid.

## Introduction

Advancements in global healthcare have facilitated the early diagnosis and timely surgical intervention of numerous thyroid diseases. Postoperative hypoparathyroidism (POHP), considered one of the most common complications following thyroid surgery, primarily results from inadvertent resection or vascular damage during the procedure, leading to acute or chronic disturbances in serum calcium and phosphate metabolism [1–3]. Furthermore, it can lead to cardiac injury, long QT syndrome, and cardiomyopathy, causing serious consequences [4]. Additionally, POHP has been linked to neurocognitive disorders, manifesting as seizure-like episodes, cognitive changes, cerebellar, and extrapyramidal symptoms [5]. Therefore, given the escalating global volume of thyroid surgeries, not only does POHP significantly impact the postoperative quality of life for a substantial number of patients, but it also has the potential to contribute to a multitude of cardiovascular, renal, and neurocognitive disorders, posing a grave threat to human health and well-being.

Currently, the main approach to POHP treatment focuses on the symptomatic management of hypocalcemia. However, prolonged and high-dose calcium and vitamin D3 supplementations not only increase the economic burden on patients and adversely affect their quality of life but also significantly disrupt calcium homeostasis, greatly elevating the risk of renal calcification and kidney stone formation [6]. Recombinant human parathyroid hormone (rhPTH) has shown promising results in correcting hypocalcemia, but its widespread application is limited in China due to the need for daily injections and its high cost. Moreover, studies suggest an increased risk of osteosarcoma associated with rhPTH, further constraining its clinical use [7, 8]. Considering the challenges associated with the treatment of hypoparathyroidism, it is crucial to accurately identify and protect the parathyroid glands during thyroid surgery.

Currently, multiple intraoperative imaging methods for parathyroid gland visualization exist. 5-aminolevulinic acid (5-ALA) is a precursor substance of fluorescent dyes that converts into the fluorescent substance protoporphyrin IX (PpIX) within the body [9]. By utilizing specific light sources and fluorescence imaging systems, the fluorescence signal of parathyroid tissue can be observed for visualization. Although it has shown potential imaging effects in some studies, further research and validation are required to ascertain its accuracy and reliability. Additionally, the imaging effect of 5-ALA is influenced by various factors, such as dosage, time, choice of light source, and usage techniques. The uncertainty associated with these factors may impact the consistency and reliability of the imaging results [10, 11]. These factors limit the application of 5-ALA in thyroid surgeries. Nano-carbon and methylene blue are two dyes used for intraoperative negative imaging of parathyroid glands, aiding in their easier identification during surgery [12, 13]. Nano-carbon may generate black deposits in the tissue, somewhat affecting the surgical field. Methylene blue, besides its impact on the surgical field, may experience a weakening of its imaging effect over time, especially during long-duration surgeries, and some patients may exhibit allergic reactions or other adverse effects to it. Therefore, the development of new imaging methods is particularly important.

Parathyroid autofluorescence is a surgical technique that utilizes near-infrared imaging to identify and locate parathyroid glands. Parathyroid tissue naturally emits fluorescence, which can be captured by a near-infrared camera [14, 15]. This technique offers a range of advantages in parathyroidectomy. It reduces the unnecessary removal of parathyroid glands and the associated risk of hypocalcemia by providing more precise localization [16]. Additionally, since it eliminates the need for contrast agents, there is a decreased risk of allergic reactions or adverse effects in patients. Of paramount importance is the real-time feedback it provides, allowing surgeons to observe the fluorescence imaging during the procedure and access immediate information for surgical decision-making [17]. However, due to the relatively new nature of this technology, research on its effectiveness is still limited [18]. This study aims to explore the potential of utilizing parathyroid autofluorescence in thyroid surgery to reduce surgical risks.

## Methods

### Study design

From September 2021 to January 2023, two surgical experts from Gulou Hospital in Nanjing participated in this study. The surgeons have each performed over 1500 thyroidectomy surgeries annually and have more than 20 years of professional experience. The participants in this study were patients undergoing total thyroidectomy. Exclusion criteria included non-thyroid diseases or previous thyroid surgeries. Patients who did not undergo thyroid surgery, including those admitted with secondary malignant lymph nodes in the neck, were excluded. Patients who were determined to no longer require surgery after evaluation, such as those with subacute thyroiditis, were also excluded. Individuals with a history of both thyroid and parathyroid surgeries were excluded as well. With the help of a computer, patients were randomly assigned to either the standard treatment group (no intraoperative use of autofluorescence) or the autofluorescence group (intraoperative use of autofluorescence). All participants provided informed consent. This study obtained ethical approval from the Ethics Committee of Nanjing Drum Tower Hospital.

### Operating procedure

In the conventional treatment group, a meticulous dissection of the thyroid capsule was performed during the thyroidectomy procedure, with careful preservation of the parathyroid glands. In the autofluorescence treatment group, three fluorescence detection processes were carried out during the thyroidectomy procedure. The first detection occurred after opening the thyroid capsule, using the fluorescence system to examine the surgical field and locate each parathyroid gland. Subsequently, the surgery proceeded as per routine. After the thyroid was removed, the second fluorescence detection was performed to ensure the parathyroid glands were preserved in their respective locations. Finally, the third fluorescence detection was conducted on the specimen, assessing for any inadvertently excised parathyroid tissue.

### Outcomes

Hypocalcemia is defined as a serum total calcium level below 2.25 mmol/L (normal range, 2.25–2.7 mmol/L) during hospitalization. Postoperative hypocalcemia patients choose oral calcium supplements or a combination of oral calcium supplements and intravenous calcium supplementation based on the severity of their symptoms. In this study, patients did not receive preventive calcium supplementation, meaning that calcium supplementation was not administered if no hypocalcemic symptoms were present after surgery. The collected data included age, gender, BMI index, the extent of central lymph node dissection (not performed, unilateral dissection, bilateral dissection), preoperative calcium level, preoperative parathyroid hormone level, duration of surgery, postoperative calcium level, postoperative day 1 PTH level, a requirement for oral calcium supplementation, a requirement for intravenous calcium supplementation, and inadvertent excision of parathyroid glands in pathology.

### Statistical analysis

We analyzed the impact of sample size on the occurrence of parathyroid gland injury in two groups: conventional treatment and autofluorescence treatment in total patients. Parameters assessed included hypocalcemia occurrence, serum PTH levels, length of hospital stay, duration of surgery, and presence of parathyroid glands in pathology. Data analysis was performed using SPSS 26.0 (SPSS Inc., Chicago, USA). A chi-square test was used for categorical variables; a *t*-test was used for normally distributed continuous variables, and binary logistic regression was used for multivariate analysis, with statistical significance set at *p* < 0.05.

## Results

### Baseline characteristics

**Fig. 1:**
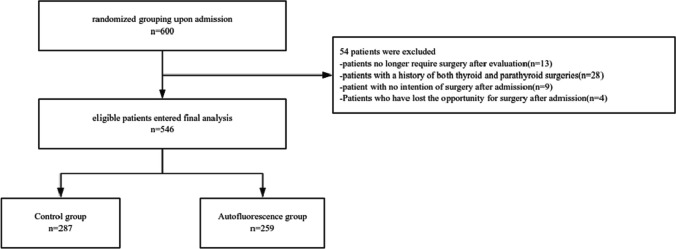
Sample selection criteria

**Fig. 2:**
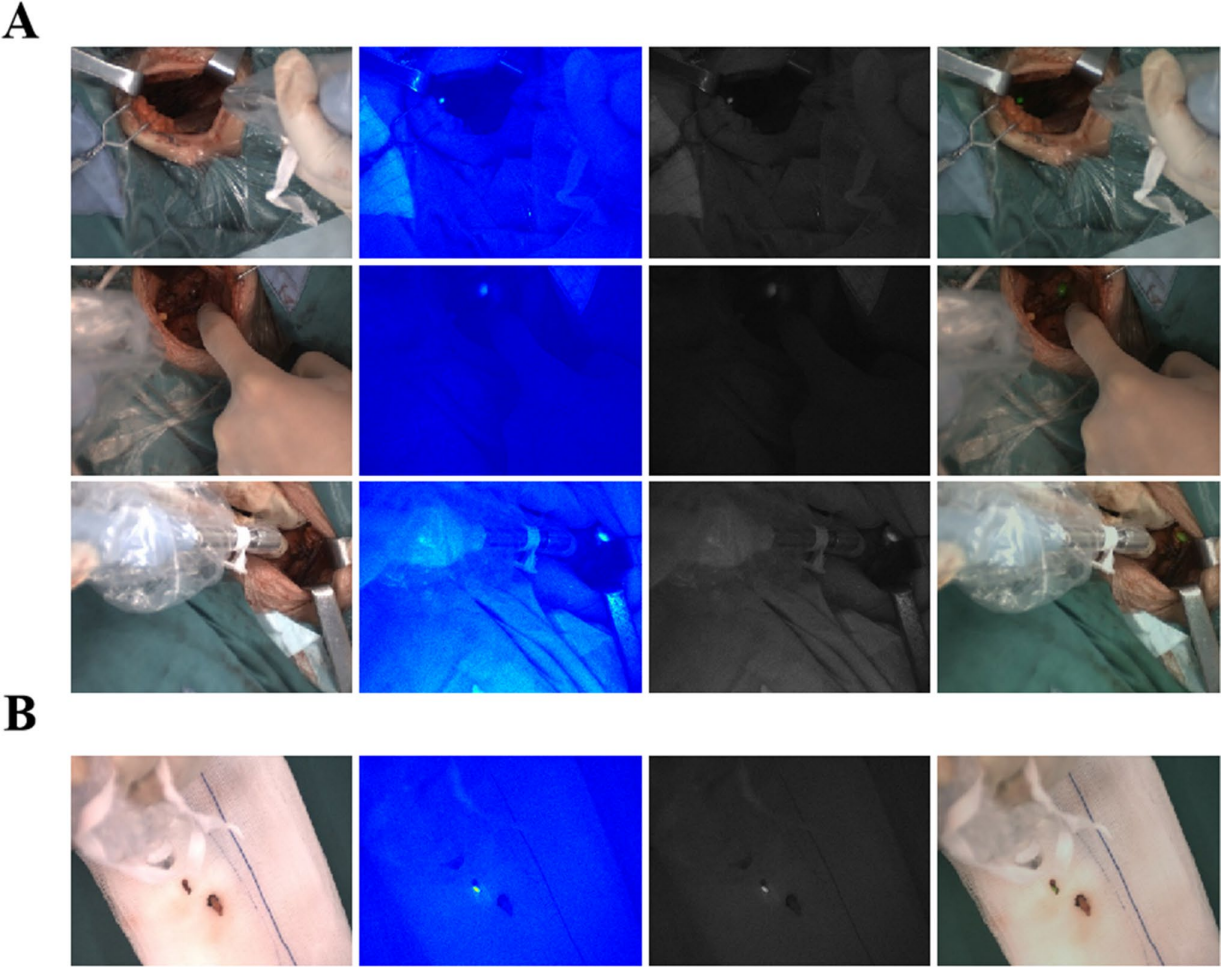
Intraoperative application of autofluorescence system facilitates postoperative parathyroid preservation. **A** Intraoperative use of parathyroid autofluorescence system for identification of parathyroid tissue, with green fluorescence indicating parathyroid tissue. **B** Examination of surgical resection specimens to detect the presence of parathyroid tissue, with green fluorescence indicating inadvertently excised parathyroid tissue during lymph node dissection, necessitating intraoperative autotransplantation

Ultimately, a total of 546 patients were included in this study, with 287 in the control group (conventional treatment) and 259 in the autofluorescence group. In the autofluorescence group, intraoperative fluorescence was used for parathyroid identification (Fig. 1A). Additionally, before specimen submission, fluorescence was employed to check for inadvertently excised parathyroid glands within the specimens (Fig. 1B). All enrolled patients underwent thyroid surgery. Baseline characteristics of patients in both groups were generally well-balanced (Fig. 2). It is worth emphasizing that numerous studies have indicated the significant impact of central lymph node dissection during thyroid surgery on postoperative parathyroid function. Therefore, we placed particular emphasis on comparing the implementation of central lymph node dissection between the two groups and found no significant differences (Table 1).

**Table 1:** Baseline characteristics of participants

Characteristic	Patients			*P*
	Usual care	Autofluorescence	Total	
Age (year)	45.82 ± 13.60	45.03 ± 11.34	45.45 ± 12.58	0.463
BMI	24.37 ± 3.72	24.126 ± 3.31	24.255 ± 3.53	0.419
Sex				
Male	106	94	200	0.877
Female	181	165	346	
Central lymph node dissection				
Yes	247	229	476	0.411
No	40	30	70	
Central lymph node dissection sides				
One side	159	150	309	0.796
Two side	88	79	167	
Preoperative serum PTH	5.279 ± 2.13	5.032 ± 2.15	5.162 ± 2.14	0.179
Preoperative serum calcium	2.40 ± 0.19	2.39 ± 0.13	2.40 ± 0.16	0.521

### Short-term outcome

An analysis of short-term outcomes for both groups of patients reveals significantly lower rates of postoperative hypocalcemia (*p* = 0.01) and postoperative parathyroid dysfunction (*p* < 0.001) in the autofluorescence group compared to the conventional treatment group. Similar results are observed for serum calcium (*p* = 0.001) and serum PTH (*p* = 0.023) levels on the first postoperative day. In response to postoperative hypocalcemia symptoms or low postoperative serum calcium levels, a therapeutic calcium supplementation strategy was employed. Upon analyzing postoperative calcium supplementation in both groups, it was evident that the autofluorescence group had significantly lower proportions of patients receiving either oral calcium supplementation (39/259) or intravenous calcium supplementation (34/259) compared to the conventional treatment group receiving oral calcium supplementation (88/287) and intravenous calcium supplementation (76/287). Contrary to our initial expectations, despite using the autofluorescence system to detect the presence of parathyroid glands in the specimens, the final pathology revealed the presence of inadvertently excised parathyroid glands in the autofluorescence group (17/259). However, this proportion was lower than that in the conventional treatment group (40/287). This discrepancy could be attributed to reduced parathyroid damage, and patients utilizing the autofluorescence system experienced a significant reduction in hospitalization duration. Surprisingly, the use of the autofluorescence system did not result in an increase in surgical time, but rather, the conventional treatment group had longer surgical durations (Table 2). To explore the impact of other factors, a multivariable, generalized, linear mixed-effects model was applied to adjust for confounding factors. It was observed that the use of autofluorescence greatly assisted in reducing the risk of postoperative parathyroid dysfunction, while age, BMI, and preoperative calcium levels showed no significant correlation with postoperative parathyroid dysfunction (Table 3).

**Table 2:** Comparison of results between the usual care group and the autofluorescence group

Characteristic	No. (%) [95% CI]		*P*
	Usual care	Autofluorescence	
Hypocalcemia	230	183	0.01
Temporary hypoparathyroidism	118	69	< 0.001
Oral calcium supplementation	88	39	< 0.001
Intravenous calcium supplementation	76	34	< 0.001
Presence of parathyroid in postoperative pathology	40	17	0.005
Serum calcium on postoperative day 1	2.10 ± 0.18 (2.08–2.12)	2.15 ± 0.16 (2.13–2.17)	0.001
Serum PTH on postoperative day 1	2.94 ± 2.40 (2.66–3.22)	3.38 ± 2.12 (3.12–3.64)	0.023
Operative time	114.22 ± 45.05 (108.98–119.45)	91.24 ± 39.81 (86.36–96.11)	< 0.001
Hospital stay	8.64 ± 2.67 (8.33–8.95)	7.51 ± 1.76 (7.29–7.73)	< 0.001

**Table 3:** Univariate and multivariate analysis for factors associated with postoperative hypocalcemia

Factor	Univariate analysis		Multivariate analysis	
	Odds ratio (95% CI)	*P*	Odds ratio (95% CI)	*P*
Use of autofluorescence	0.520 (0.362–0.747)	< 0.001	0.506 (0.349–0.733)	< 0.001
Age	1.010 (0.996–1.024)	0.170	1.011 (0.996–1.027)	0.143
Sex	1.091 (0.757–1.573)	0.640	1.126 (0.842–1.845)	0.272
BMI	0.989 (0.940–1.040)	0.645	0.971 (0.920–1.025)	0.284

### Autofluorescence impact on central lymph node dissection

**Fig. 3:**
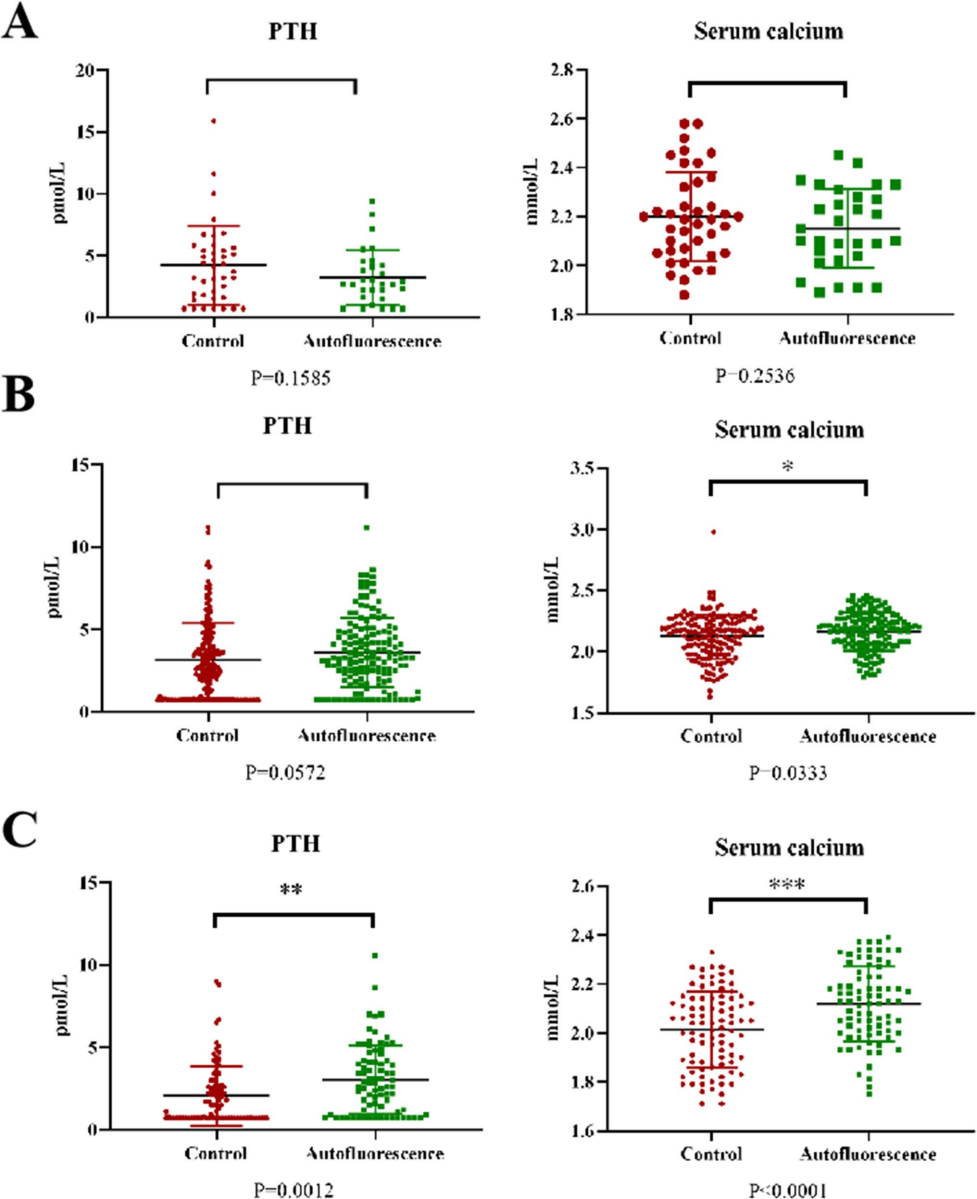
Comparison of postoperative serum calcium and PTH levels between the conventional group and the autofluorescence group in different situations. **A** Comparison of postoperative serum calcium and PTH levels between the conventional group and the autofluorescence group when no central lymph node dissection is performed. **B** Comparison of postoperative serum calcium and PTH levels between the conventional group and the autofluorescence group when unilateral central lymph node dissection is performed. **C** Comparison of postoperative serum calcium and PTH levels between the conventional group and the autofluorescence group when bilateral central lymph node dissection is performed

Numerous studies have highlighted a strong association between central lymph node dissection and postoperative parathyroid dysfunction. In this study, we conducted a detailed analysis of the data. We categorized the patients into subgroups based on whether central lymph node dissection was not performed, performed on one side, or performed bilaterally. Our results revealed that the use of autofluorescence in patients who did not undergo central lymph node dissection did not significantly affect postoperative serum calcium and PTH levels (Fig. 1C). However, when the autofluorescence system was employed in patients undergoing unilateral central lymph node dissection, although the serum PTH levels did not show statistical differences (*p* = 0.0572), there was a significant difference in serum calcium levels (*p* = 0.0333) (Fig. 1D). Remarkably, in patients undergoing bilateral central area clearance, the autofluorescence system exhibited substantial advantages, leading to significant enhancements in both serum calcium levels (*p* = 0.0012) and PTH levels (*p* < 0.0001) due to its utilization as shown in Fig. 3C.

## Discussion

The parathyroid glands, four small glands located behind the thyroid, are responsible for regulating calcium and phosphate levels in the body [19, 20]. Accurately identifying and protecting these glands during thyroid or parathyroid surgery is of paramount importance, as their damage or inadvertent removal can lead to severe disruptions in calcium metabolism. We have observed that the use of the autologous fluorescence system, which was developed to precisely and in real-time locate the parathyroid glands [21, 22], can reduce the likelihood of post-thyroid surgery parathyroid gland injuries, thereby lowering the risk of intraoperative misexcision and subsequently reducing the occurrence of postoperative hypocalcemia due to parathyroid gland functional decline [23, 24]. In our study, the utilization of the fluorescence system effectively decreased the probability of parathyroid gland involvement in postoperative pathology, indicating its efficacy in reducing the likelihood of parathyroid misexcision. As previous research reported by Benmiloud et al. [16], the utility of autofluorescence systems in thyroid surgery was confirmed. Considering that most studies support the connection between central lymph node dissection and parathyroid function impairment, we observed the outcomes of applying autofluorescence systems in populations with varying degrees of central lymph node dissection [25]. We categorized patients based on the extent of central lymph node dissection, including those who did not undergo central lymph node dissection, those who had one-side central lymph node dissection, and those who had bilateral central lymph node dissection. Our results demonstrated that the autologous fluorescence system holds greater value for patients requiring bilateral central lymph node dissection. For patients not undergoing central lymph node dissection, the utility of the autologous fluorescence system was not as significant as initially anticipated.

Although parathyroid autofluorescence near-infrared imaging technology has brought numerous benefits to parathyroid surgery, its application still exhibits certain limitations. Firstly, the high equipment cost of near-infrared imaging systems and their associated devices has hindered the adoption of this technology by some healthcare institutions [26–28]. While near-infrared imaging can aid physicians in identifying parathyroid glands more easily, there remains a risk of identification errors, particularly in cases involving lesions or abnormal anatomical structures. Additionally, limitations may arise when imaging deep-seated tissues, and there may be shortcomings in displaying parathyroid glands covered by the connective tissue membrane [29, 30]. Currently, the near-infrared system is influenced by the surgical environment, as evident from our intraoperative observations where electrocautery-induced eschar may interfere with fluorescent signals, consequently affecting imaging quality. Despite these limitations and drawbacks, parathyroid autofluorescence near-infrared imaging continues to be considered a valuable tool in parathyroid surgery, especially in complex or high-risk procedures. With ongoing technological advancements and increased clinical research, we can anticipate that these issues will be addressed in the future.

In summary, despite some minor defects in the current autologous fluorescence system, these problems will eventually be overcome with technological progress. Thanks to its non-invasion and excellent performance in parathyroid recognition and protection, we believe that it will become an indispensable and important technology in thyroid surgery in the future.
