# Correction to: The application of autofluorescence system contributes to the preservation of parathyroid function during thyroid surgery

**DOI:** 10.1007/s00423-024-03580-w

**Published:** 2024-12-27

**Authors:** XianBiao Shi, Guan Lv, JiaBo Qin, Yixuan Li, Lulu Zheng, Haoran Ding, JianFeng Sang

**Affiliations:** 1 Nanjing Drum Tower Hospital, Nanjing, China; 2 Nanjing Drum Tower Hospital Clinical College of Nanjing Medical University, Nanjing, China; 3 Nanjing Medical University, Nanjing, China; 4 Nanjing University, Nanjing, China

**Correction to: Langenbeck’s Archives of Surgery (2024) 409:96**

10.1007/s00423-024-03256-5

In this article, Guan Lv was affiliated with ‘Nanjing Medical University, Nanjing, China’ and JianFeng Sang was affiliated with ‘Nanjing Drum Tower Hospital, Nanjing, China’. The correct affiliation of both authors should be ‘Nanjing Drum Tower Hospital Clinical College of Nanjing Medical University, Nanjing, China’.

The original article has been corrected.

